# Digital health tools for pain monitoring in pediatric oncology: a scoping review and qualitative assessment of barriers and facilitators of implementation

**DOI:** 10.1007/s00520-023-07629-2

**Published:** 2023-02-21

**Authors:** J. D. H. P. Simon, I. S. Hooijman, M. Van Gorp, S. A. Schepers, E. M. C. Michiels, W. J. E. Tissing, M. A. Grootenhuis

**Affiliations:** 1 Princess Máxima Center for Pediatric Oncology, Heidelberglaan 25, 3584 CS, Utrecht, The Netherlands; 2 Department of Pediatric Oncology, University of Groningen, University Medical Center Groningen, Groningen, The Netherlands

**Keywords:** Pain, Pediatric oncology, Digital health, mHealth, Implementation

## Abstract

**Purpose:**

We aimed to systematically identify and characterize existing digital health tools for pain monitoring in children with cancer, and to assess common barriers and facilitators of implementation.

**Methods:**

A comprehensive literature search (PubMed, Cochrane, Embase, and PsycINFO) was carried out to identify published research on mobile apps and wearable devices focusing on acute and/or chronic pain in children (0–18 years) with cancer (all diagnoses) during active treatment. Tools had to at least include a monitoring feature for one or more pain characteristic(s) (e.g., presence, severity, perceived cause interference with daily life). Project leaders of identified tools were invited for an interview on barriers and facilitators.

**Results:**

Of 121 potential publications, 33 met inclusion criteria, describing 14 tools. Two methods of delivery were used: apps (*n*=13), and a wearable wristband (*n*=1). Most publications focused on feasibility and acceptability. Results of interviews with project leaders (100% response rate), reveal that most barriers to implementation were identified in the organizational context (47% of barriers), with financial resources and insufficient time available mentioned most often. Most factors that facilitated implementation related to end users (56% of facilitators), with end-user cooperation and end-user satisfaction mentioned most often.

**Conclusions:**

Existing digital tools for pain in children with cancer were mostly apps directed at pain severity monitoring and little is still known about their effectiveness. Paying attention to common barriers and facilitators, especially taking into account realistic funding expectations and involving end users during early stages of new projects, might prevent evidence based interventions from ending up unused.

**Supplementary Information:**

The online version contains supplementary material available at 10.1007/s00520-023-07629-2.

## Introduction

Children with cancer experience a wide range of symptoms as a result of their illness and/or treatment [1]. These symptoms include pain, nausea, and fatigue [2]. As survival rates of pediatric cancer improve [3, 4], the focus on supportive care (i.e., the management and prevention of adverse symptoms of the illness and its treatment) has increased [5]. Pain is one of the most common adverse symptoms during childhood cancer treatment with prevalence rates varying between 40 and 78% [2, 6–11]. It is also the symptom most feared by children [12]. Cancer-related pain is often caused by the treatment (chemotherapy, surgery, or radiation), by procedures (lumbar punctures, blood draws, or bone marrow aspirations), or by the illness itself (tumor infiltration in tissues or organs) [10, 13].

A previous study on pain in children receiving chemotherapy at the outpatient clinic showed that the majority (78%) experienced clinically significant pain (score ≥ 4), some even more than half of the time (30%) [9]. In one-third (33%) of the clinically significant pain incidences reported in this study, no interventions were used to reduce the pain. This might be due to parental misconceptions (e.g., pain in cancer is inevitable) [10] or concerns regarding analgesic use in children (e.g., pain medication is addictive and works best when used as little as possible) [14]. Despite existing evidence for a variety of effective pain prevention and pain management strategies [15], the management of pain in the home setting is still suboptimal [9]. Digital health provides healthcare organizations with an opportunity to bridge the distance between the hospital and home setting, and to offer support remotely. Digital health includes electronic health (eHealth) and mobile health (mHealth) [16] and has many potential benefits such as accessibility and availability to a wider public (anywhere, anytime), the ability to provide real-time strategies in everyday settings, and to fine-tune interventions to end users’ individual needs [17].

Over the years, the amount of digital health tools for pain management has grown rapidly [18–21]. The range of features used in existing digital tools for pain seems to vary widely, from more basic tools providing information about pain(management) or using symptom diary tracking, to more advanced tools including real-time feedback from healthcare professionals (HCPs) and game elements (i.e., gamification) such as personalized avatars or in-app rewards to increase user engagement and motivation to symptom reporting [22, 23]. The literature shows growing evidence for the feasibility, acceptability, and effectiveness of digital tools in adult cancer patients and survivors [19, 24]. In the pediatric oncology population, the feasibility and acceptability of some digital tools for cancer-related symptoms, including pain, have been assessed as well [25], yet results on their effectiveness are scarce. One systematic review looked at the effectiveness and efficacy of digital health tools for children and young adults undergoing cancer treatment and survivors [25]. The results of the two identified studies examining the impact on pain were mixed: one study using virtual reality did not demonstrate a significant change in self-reported pain intensity [26], and one study using an app did [27], yet this was a pilot study with preliminary results.

The rapid development and rise of these, often very costly, tools raise the urgency of implementation science [18]. It generally takes approximately 15–20 years to successfully implement a new evidence-based intervention in healthcare settings [28, 29], and only 14% of interventions are successfully adopted in routine care [29], resulting in a large amount of “research waste.” Implementation science uses strategies to adopt and integrate evidence-based health interventions into a clinical setting and describes “the effects of deliberate and purposive actions to implement new treatments, practices, and services” [29]. In order to prevent evidence-based interventions from ending up unused, it is imperative to assess and address determinants that are slowing down (i.e., barriers) and/or facilitating (i.e., facilitators) implementation [30]. In order to make optimal use of this knowledge and focus on areas that need more attention, barriers, and facilitators need to be identified at an early stage. A review on the availability of pain-related eHealth interventions in routine pediatric care found researchers’ intrinsic motivation (i.e., personal beliefs in the importance of making their tools available to end users) to be the most endorsed facilitator, whereas system-level issues (e.g., lack of time and infrastructure to support intervention availability) were common barriers [30]. Including end users in the design phase (user-centered design) was associated with intervention availability in routine care [30]. This is consistent with other reviews on digital tools stressing the importance of involving key stakeholders throughout the entire process to attain buy-in from these parties [25, 31–33]. Stakeholders are defined as all people and/or organizations that affect or are affected by the outcomes of a project [34].

In children with cancer, pain has been identified as one of the most common symptoms during all phases of cancer treatment (acute as well as follow-up). Relative to other pediatric diagnoses, their treatment is particularly intense and toxic. Moreover, with new treatment regimens allowing patients to spend more time at home, the responsibility of managing pain lies with families themselves more than ever [35, 36]. Therefore, there is a need to identify digital health tools aimed at the pediatric population specifically, as these might help parents and children cope better. Two systematic reviews (2020) reported on the availability of digital health tools for cancer-related symptoms in pediatric patients [20, 25], yet in both studies, only a limited number (*n*=2) of tools aimed at pain were identified. As the field of digital health is still rapidly evolving, we expect that an update on the subject will yield more results.

Moreover, this review will focus on mobile applications (“apps”) and wearable devices specifically. The reasoning behind this is that we want to include digital health tools that are always at hand and enable real-time (i.e., prospective) pain assessments, in order to avoid recall bias [37]. Thus, we aim to identify and characterize existing digital health tools (i.e., mobile apps and wearable devices) for pain in children with cancer. For each tool, we aim to provide an overview of research findings (e.g., feasibility, acceptability, effectiveness), and to assess common barriers and facilitators (i.e., lessons learned). By doing so, we hope to gain insight into existing digital tools for pain in pediatric oncology specifically, and secondly to compile valuable lessons for future digital health developers and researchers, not only in pediatric oncology but in a broader range of pediatric healthcare settings.

## Methods

### Design and reporting

We used Arksey and O’Malley’s methodological framework for scoping reviews to examine the extent, range, and nature of research activity, and to summarize and disseminate our research findings [38]. The framework consists of (*step 1*) identifying an aim, (*step 2*) identifying relevant studies (i.e., carry out a literature search), (*step 3*) selecting studies based on inclusion and exclusion criteria, (*step 4*) charting the data, and (*step 5*) collating, summarizing, and reporting the results and is in accordance with the extended PRISMA guideline for Scoping Reviews [39]. No review protocol exists for the current review.

### Search strategy and eligibility criteria

A search strategy was created with a medical librarian and carried out on February 9th 2022. Eligible publications were identified through searches of PubMed, Cochrane, Embase, and PsycINFO. The search consisted of four main search terms (ehealth/mhealth, pain, children, and cancer), each consisting of multiple keywords. A detailed overview of the included keywords and search string used for PubMed can be found in Appendix 1. Medical Subject Headings (MeSH), or equivalent terms, were used. No date range was used to limit the search and only English publications were considered. Additional publications were manually searched by scanning reference lists of identified publications.

We included publications (*a*) concerning mobile apps and wearable devices aimed at pain (*b*) with (at least) a monitoring feature for one or more pain characteristic(s) (e.g., presence, severity, perceived cause, interference with daily life), (*c*) for children with a cancer diagnosis (all diagnoses) (*d*) aged between 0 and 18 years old (or their parents) (*e*) during active treatment.

The literature management program EndNote was used to remove duplicates, after which the remaining publications were transported into Rayyan [40], which was used to remove the remaining duplicates and to enable multiple authors to screen the publications independently. Two reviewers (JDHPS and ISH) screened the publications independently for eligibility based on abstract and title. Disagreements were resolved by discussion and consensus. A final review of full-text versions of the selected publications was carried out by JDHPS and ISH to determine eligibility.

### Semi-structured interviews

The second objective was to assess the determinants of implementation of the identified tools. For this purpose, the corresponding authors of these tools were approached for a semi-structured interview about their tool via a live video communication platform (Zoom). The interviews were audio-recorded after obtaining permission from the interviewees and consisted of three sections: (1) current project phase and parties involved (i.e., professionals who contributed to the project and key stakeholders), (2) use of implementation theory/model/framework, (3) and key barriers and facilitators encountered during the project. Finally, demographic information and working experience of interviewees were collected.

The Measurement Instrument for Determinants of Innovations (MIDI) was used to guide section 3 of the interview (key barriers and facilitators encountered during the project) [41]. The MIDI categorizes barriers and facilitators into 4 main themes and 29 subthemes. The main themes are a tool (e.g., complexity, compatibility), end user (e.g., personal benefits/drawbacks, satisfaction), organization (e.g., formal ratification by management, replacement when staff leave), and socio-political context (e.g., legislation and regulations). We added an additional sub-theme to socio-political context (collaborating with external stakeholders, i.e., other disciplines/hospitals/cultures) since external collaboration barriers/facilitators were not included in the MIDI. An overview of the MIDI (sub)themes was sent to the interviewees prior to the interview and was displayed during the interview when barriers and facilitators were discussed [41]. After a barrier/facilitator was mentioned by an interviewee, the interviewer and interviewee collaboratively categorized it into a corresponding MIDI theme and subtheme. The interview guide, including the overview of MIDI (sub)themes, can be found in Appendix 2.

### Data charting and synthesis of results

The data from the publications identified in the literature search was charted by giving an overview of tool characteristics based on published research, namely method of delivery, features, end users, and published research (including outcome measures and main findings) in table form. Published studies were categorized by study design based on what was reported in articles. During interviews, aspects that remained unclear based on published research were verified with the project leaders. Finally, all project leaders were requested to verify the data in the table via email (Table 1).

**Table 1:** Identified tools, characteristics, and published research (*N*=14 tools)

Digital tool	Tool characteristics	Publications (*N*=33 included in total)	
**Color Me Healthy app** *Main goal:* To acquire child-centric symptom reports	Method of delivery • Mobile application (tablet) Feature(s) • *Pain monitoring (among other symptoms):* - Severity (4-point Likert scale) • *Game-element(s):* - Users can pick/customize their avatar - Sketch pad to locate pain End-user(s) • Children with cancer and one of their parents • Age: 6−12 • Inpatients and outpatients	Feasibility/acceptability study ( *N* =19 patients) (2020) [ 1]	
		**Outcome measures:** • Acceptability • Feasibility	**Main finding(s):** Preliminary acceptability and feasibility established
		Secondary analysis: self-reports of pain ( *N* =19 patients) (2021)[2]	
		**Outcome measures:** • Pain frequency • Pain severity • Pain bother • Pain location	**Main finding(s):** 100% reported pain at least once. Most frequent reported severity: ‘mild’. Most frequent reported bother: ‘mild’. Most frequent reported location: ‘head’
		Secondary analysis: parental feedback ( *N* =19 parents) (2022)[3]	
		**Outcome measures:** • Parental perceived benefits	**Main finding(s):** Parents perceived tool to 1) elicit child’s voice about symptom experience, 2) provide supportive/safe environment for child to report symptoms, 3) create opportunity to facilitate communication between child, parent, and clinical team
		Descriptive study ( *N* =19 patients) (2022)[4]	
		**Outcome measures:** • Reported symptoms • Reported daily experiences	**Main finding(s):** 100% reported symptoms at least once, and *n*=14 reported at least one day with symptom of moderate or higher severity
**Computerized Symptom Capture Tool (C-SCAT)** *Main goal:* To better understand symptom experiences of patients	Method of delivery • Mobile application (tablet) Feature(s) • *Pain monitoring (among other symptoms):* - Pain presence (Memorial Symptom Assessment Scale [MSAS], adult version) - Severity (4-point Likert scale) - Interference with daily life (5-point Likert scale) - Perceived causes (free text) - Alleviating and exacerbating factors (free text) • *Communication:* - Discuss reported scores during planned clinic visit End-user(s) • Adolescents and Young Adults (AYA’s) with cancer • Age: 13−29 • Inpatients and outpatients	Feasibility/acceptability study ( *N* =72) (2014)[5]	
		**Outcome measures:** • Feasibility • Acceptability • Reported symptoms • Reported symptom clusters	**Main finding(s):** Tool demonstrated feasibility and acceptability
		Mixed-methods descriptive study ( *N* =72) (2015)[6]	
		**Outcome measures:** • Experienced symptoms • Symptom clusters	**Main finding(s):** Most frequently reported symptoms were nausea, feeling drowsy, lack of appetite, and lack of energy. The most common symptom cluster was nausea/eating problems/ appetite problems
		Descriptive study ( *N* =72) (2017)[7]	
		**Outcome measures:** • Self-management strategies used	**Main finding(s):** 772 reported self-management strategies were organized into three overarching themes (and 16 sub themes): “Things I take..or not”, “Physical care things I do”, and “Psychosocial care things I do”. Medications was the most frequently reported strategy
		Descriptive study ( *N* =86) (2019)[8]	
		**Outcome measures:** • Frequency and characteristics of priority symptoms • Self-management strategies used for priority symptoms	**Main finding(s):** Lack of energy, nausea, difficulty sleeping, and pain comprised 39% of all (189) priority symptoms. Self-management strategies included “Physical care strategies”, “Things I take (or not)”, and “Psychosocial care strategies”
		Effectiveness study ( *N* =88) (2019)[9]	
		**Outcome measures:** • Effect on self-efficacy for symptom management and self-regulation related to symptoms • Communication with HCP’s	**Main finding(s):** 85% showed improved self-efficacy for managing symptoms. Qualitative data suggest usefulness for enhancing self-regulation abilities. AYA’s reported that tool facilitated communication with HCP’s about symptom (management).
		Feasibility study ( *N* =86) (2020)[10]	
		**Outcome measures:** • HCP’s perceptions of usefulness • HCP’s perceptions of ease of use	**Main finding(s):** Use of tool enhanced HCP’s understanding of AYA’s symptom experiences
		Descriptive study ( *N* =118) (2021)[11]	
		**Outcome measures:** • Symptom experiences of AYA’s across five cancer diagnostic groups based on individual factors	**Main finding(s):** Across diagnostic groups, symptoms varied little based on individual factors. This supports a heterogeneous approach to symptom research with AYA’s
**Empatica E4 wristband** *Main goal:* To provide more effective individualize care to patients	Method of delivery • Wearable device (wristband) Feature(s) • *Pain monitoring:* - Heart rate - Skin temperature - Electro dermal activity End-user(s) • Children with cancer and sickle cell disease, admitted for acute/chronic pain • Age: 7−20 • Inpatients	Feasibility/acceptability study ( *N* =12) (2021)[12]	
		**Outcome measures:** • Ability of wireless device to obtain vital signs • Children’s perception of wristband	**Main finding(s):** Data collected with tool correlated with manually obtained vital signs. Children responded favourable to wearing the tool
**ePROtect** *Main goal:* To acquire symptom self-reports for a more patient-directed cancer control approach	Method of delivery • Mobile application (phone and tablet) Feature(s) • *Pain monitoring (among other symptoms):* - Severity (smiley scale three faces (< 8 years), 5-point Likert scale (> 8 years) - Interference with daily life (5-point Likert scale) • *Communication:* - Real-time feedback from Healthcare Professionals (HCP’s) End-user(s) • Children with cancer being treated with chemotherapy • Age: 0−18 • Inpatients and outpatients	Feasibility study ( *N* =12) (2021)[13]	
		**Outcome measures:** • Patient satisfaction • Usefulness • Completion rate and time • Symptom prevalence • Frequency of interventions based on patient-reported outcome measures (PROMs)	**Main finding(s):** Over 80% of patients/proxies provided feedback with a high rating for satisfaction and usefulness of tool. The median percentage of completion days was 85.3%, and mean time to complete was 47.6 seconds. Severe symptoms were reported in 14.7% of measurements, which led to interventions in 57 cases
**KLIK Pain Monitor** **app** *Main goal:* To improve pain management in the home setting	Method of delivery • Mobile application (phone and tablet) Feature(s) • *Pain monitoring:* - Severity (Numerical Rating Scale [NRS]-11) • *Information about pain (management)* • *Communication:* - Real-time feedback from HCP’s End-user(s) • Children with cancer • Age: 0−18 • Outpatients	Feasibility study ( *N* =27) (2021)[14]	
		**Outcome measures:** • Family adherence • HCP adherence • Feasibility • Barriers and facilitators of implementation	**Main finding(s):** 18.5% Of families reported pain twice daily (family adherence). In 70% of high pain scores, HCP’s followed-up within set timeframe (HCP adherence). The majority of app functions are feasible. Facilitators related to user friendliness, and barriers related to technical problems with daily reminders
**Kræftværket app** *Main goal:* To create a virtual community that empowers patients and improves their quality of life	Method of delivery • Mobile application (phone) Feature(s) • *Pain monitoring (among other symptoms):* - Severity (smiley scale five faces) • *Information about pain (management)* • *Communication:* - Community forum End-user(s) • AYA’s with cancer and during follow-up • Age: 16−32 • Inpatients and outpatients	Prototype development study ( *N* =17) (2018)[15]	
		**Outcome measures:** • User-involved development of prototype app	**Main finding(s):** Three key features to be included in prototype were identified during co-creation workshops: 1) community forum, 2) information library, 3) symptom and side-effect tracking tool
		Usability study ( *N* = 20) (2020)[16]	
		**Outcome measures:** • Usability of prototype based on think-aloud test	**Main finding(s):** AYA cancer apps should be relevant, targeted, and unique, and include a tracking function and AYA-produced videos
		Feasibility study ( *N* =17) (2021)[17]	
		**Outcome measures:** • Patient reported relevance • Patient reported usefulness	**Main finding(s):** Tool was perceived most relevant at disease onset. During treatment, diagnosis-specific information and communities were requested
**Mobile Oncology Symptom Tracker (mOST)** *Main goal:* To record symptoms in real-time	Method of delivery • Mobile application (phone) Feature(s) • *Pain monitoring (among other symptoms):* - Severity (Faces Pain Scale Revised [FPS-R], Color Analog Scale [CAS]) End-user(s) • AYA’s with cancer • Age: 13−21 • Inpatients and outpatients	Usability/acceptability study ( *N* =10) (2012)[18]	
		**Outcome measures:** • Patient adherence • Usefulness and acceptability	**Main finding(s):** Adherence to daily symptom reports exceeded 90%. Patients experienced few technical difficulties and reported benefits from daily symptom reports
**OPBG (Ospedale Pediatrico Bambino Gesu Tool)** *Main goal:* To track pain in the home setting	Method of delivery • Mobile application (phone and tablet) Feature(s) • *Pain monitoring:* - Severity (Combination between the NRS-11, FPS-R, CAS, VAS): sliding from left to right the face gradually changes expression until it becomes a crying face at the score of 10 End-user(s) • Children with cancer or their parents • Age: 4−21 • Outpatients	Adherence to use and pain prevalence study ( *N* =124) (2021)[19]	
		**Outcome measures:** • Pain levels • App use • Patient satisfaction	**Main finding(s):** 75.8% reported pain (>1) at least once during one month, of these, 56.4% reported mild pain, 35.1% moderate pain and 8.5% severe pain. 124 participants used the app for at least a month. The median number of times participants used the app during that time was 6. Most participants were satisfied with app
**Pain Buddy** *Main goal:* To enhance pain management and foster improved quality of life during cancer treatment	Method of delivery • Mobile application (phone and tablet) Feature(s) • *Pain monitoring:* - Pain presence (MSAS, yes/no) - Severity (VAS, 0−100) - Frequency (4-point Likert scale) - Bothersomeness (5-point Likert scale) - Pain location (Adolescent Pediatric Pain Tool [APPT], VAS, 0-100) - Intensity (word graphic rating scale) - Quality (list of pain descriptors) • *Information about pain (management)* • *Communication:* - Real-time feedback from HCP’s through web-interface • *Gamification:* - Users can pick their three-dimensional avatar - Adherence-based reward system End-user(s) • Children with cancer • Age: 8−18 • Outpatients	Pilot development study ( *N* =12) (2016)[20]	
		**Outcome measures:** • Overview of development phase • Feasibility • Preliminary outcome data	**Main finding(s):** Key aspects: daily pain and symptom diaries, remote monitoring, cognitive and behavioral skills training, interactive avatars, and an incentive system to motivate engagement. Children were highly satisfied. Pain and appetite disturbances were most frequently endorsed
		Preliminary effectiveness study ( *N* =48) (2020)[21]	
		**Outcome measures:** • Reduction in average daily pain over study period (intervention vs control)	**Main finding(s):** Both groups experienced significant reductions, with no evident group differences. Intervention group did report significantly fewer instances of moderate to severe pain compared with control group
**Pain Squad app** *Main goal:* To assess pain in real-time during cancer treatment	Method of delivery • Mobile application (phone and tablet) Feature(s) • *Pain monitoring:* - Severity (VAS, 0−10) - Interference with daily life (VAS, 0−10) - Pain management control (VAS, 0−10) • *Gamification:* - Users play the role of law-enforcement officers - Adherence-based reward system End-user(s) • Children with cancer • Age: 8−18 • Inpatients and outpatients	Development, usability and feasibility study ( *N* =47) (2013)[22]	
		**Outcome measures:** • Usability • Feasibility • Compliance • Satisfaction	**Main finding(s):** App was overall appealing to patients. They endorsed game-based nature of app and its virtual reward system. Compliance was high (mean 81%) and patients found app to be likeable, easy to use, and not bothersome to complete
		Psychometric validation study ( *N* =106) (2015)[23]	
		**Outcome measures:** • Construct validity • Reliability • Feasibility	**Main finding(s):** Results provide evidence of construct validity, reliability, and feasibility of tool
		Pilot implementation study ( *N* =6) (2018)[24]	
		**Outcome measures:** • Implementation outcomes (acceptability, appropriateness, cost, feasibility, fidelity, penetration, and sustainability)	**Main finding(s):** Tool was well received by small number of children, yet user uptake, engagement, and adherence were significant barriers to implementation in a natural setting
**Pain Squad + app** *Main goal:* To assess pain in real-time and provide patients with real-time pain management support	Method of delivery • Mobile application (phone and tablet) Feature(s) • *Pain monitoring:* - Severity (VAS, 0−10) - Interference with daily life (VAS, 0−10) - Pain management control (VAS, 0−10) • *Information about pain(management)* • *Communication:* - Real-time, algorithm informed feedback • *Gamification:* - Users play the role of superheroes - Adherence-based reward system End-user(s) • Children with cancer • Age: 12−18 • Inpatients and outpatients	Development study ( *N* = 9 HCP, 10 patients) (2014)[25]	
		**Outcome measures:** • Pain management advice algorithm • System design requirements	**Main finding(s):** A systematic literature review and 2-day consensus conference established which clinically important pain inputs would require action (pain management advice) from tool, the appropriate advice the tool should provide and the functional requirements of the tool
		Iterative usability testing study ( *N* =16) (2017)[26]	
		**Outcome measures:** • Ease of use • Ease of understanding • Efficiency • Acceptability	**Main finding(s):** Patients required an average of 4.3 minutes to complete pain assessment. Overall, the tool was acceptable. Problematic issues related to software malfunction, interface design flaws and confusing text
		Implementation and preliminary effectiveness study ( *N* =40) (2017)[27]	
		**Outcome measures:** • Intervention fidelity (implementation outcome) • Outcome measure completion • Adherence • Acceptability • Preliminary effectiveness	**Main finding(s):** Intervention fidelity was impacted by technical difficulties, and a prolonged time for HCP contact in event of sustained pain. Outcome measure completion rates were high and tool was acceptable. Trends in improvements in pain intensity, pain interference, and Health Related Quality of Life (HRQL) were significant
		Nested qualitative study within multicenter pilot feasibility study ( *N* =20) (2018)[28]	
		**Outcome measures:** • Acceptability • Perceived helpfulness • Suggestions for improvement • Satisfaction with pilot study protocol	**Main finding(s):** Overall, the tool was acceptable to patients. Suggestions for tool and study improvements were identified and will be incorporated into future RCT design
		Protocol for RCT ( *N* =74 per arm; *arm 1= control waitlist, 2= app with nurse, 3 = app no nurse)* (2020)[29]	
		**Outcome measures:** • Effect on pain intensity • Effect on pain interference, HRQOL, pain self-efficacy and cost • Satisfaction with treatment regarding tool	**Main finding(s):** Not applicable: research protocol
**PicPecc** **(Pictorial support in person-centred care for children)** *Main goal:* To acquire self-reports of symptoms experienced by ALL patients	Method of delivery • Mobile application (phone and tablet) Feature(s) • *Pain monitoring (among other symptoms):* - Severity (Faces Thermometer Scale [FTS]) • *Information about pain(management)* • *Communication:* - Real-time feedback HCP’s and visual feedback via app (pictorial support) End-user(s) • Children with ALL, undergoing high-dose methotrexate treatments • Age: 5-17 • Inpatients and outpatients	Protocol for crossover design study (2021)[30]	
		**Outcome measures:** • Distress levels (intervention vs control group) • Level of person-centered care • Symptom assessment frequency	**Main finding(s):** Not applicable: research protocol
		Development study ( *N* =7 children, 8 parents, 19 HCP [phase 1], *N* = 10 children, 9 parents, 21 HCP [phase 2] (2022)[31]	
		**Outcome measures:** • Symptom-reporting needs • Evaluation of patients’ tool interaction • Parents’ views and HCP’s expectations and requirements for tool	**Main finding(s):** Various symptom-reporting needs were identified. The findings of this study indicate that the tool is a potential solution for providing communicative support to patients. Interview data also highlighted symptoms that are at risk of being overlooked if not included in the tool
**Telemonitoring System for Paediatric Oncology** *Main goal:* To increase the quality of life of patients during cancer treatment by monitoring symptoms in the home setting	Method of delivery • Mobile application (phone) Features(s) • *Pain monitoring* (*among other symptoms*)*:* - Severity (6-point Likert scale) • *Communication:* - Web-interface for HCP’s to evaluate data End-user(s) • Children with cancer • Age: 0-18 • Outpatients	Development/feasibility study (2015) [ 32]	
		**Outcome measures:** • Acceptability • Telemonitoring workflow	**Main finding(s):** Nine vital signs and toxicities have been identified as most significant to monitor in pediatric cancer patients. Experiences from the user centred design process indicate a high level of usability and acceptability
**RESPONSE** *Main goal:* To monitor symptoms in children undergoing systemic cancer treatment	Method of delivery • Mobile application (phone and tablet) Features(s) • *Pain monitoring (among other symptoms)* - Bothersomeness (5-point Likert scale) • *Information about pain(management)* • *Communication:* - Web-interface for HCP’s to evaluate data - Algorithm-informed alerts advising families to discuss with HCP at next hospital visit (moderate concern), or phone hospital (immediate concern) End-user(s) • Parents of children with blood cancer and solid tumors (or adolescents themselves) • Age: 2−18 • Inpatients and outpatients	Protocol for controlled hybrid effectiveness implementation trial (2021)[33]	
		**Outcome measures:** • Effectiveness on total symptom burden of children • Effects on health-related quality-of-life • Feasibility • Acceptability • Satisfaction • Sustainability	**Main finding(s):** Not applicable: research protocol

The outcomes of the interviews on barriers and facilitators were summarized based on audio recordings and represented in table form and visualized in graphs. Each interviewee was requested to verify the data in the table (Appendix 3).

## Results

### Publication selection process

**Fig. 1:**
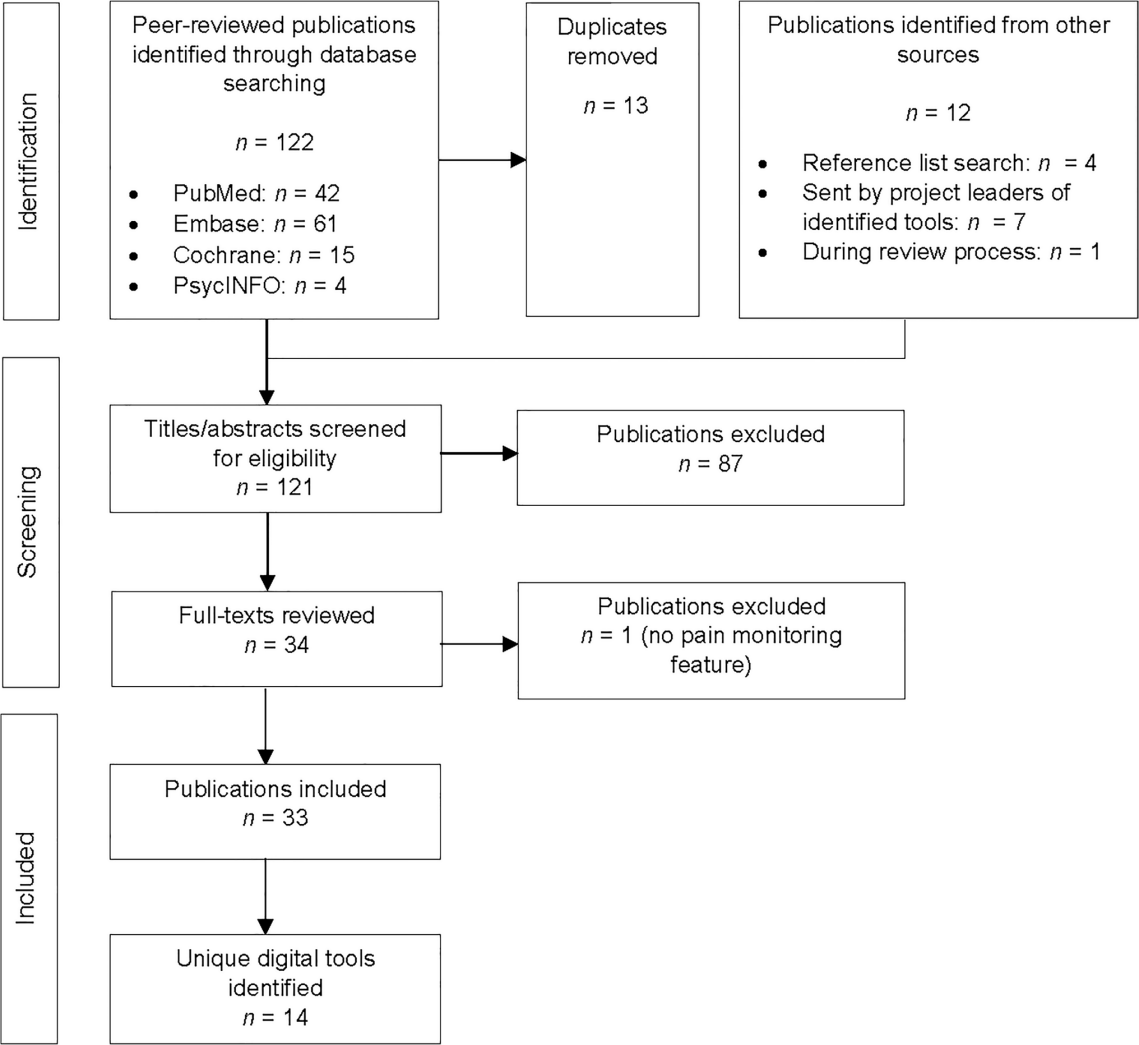
Flow diagram scoping review

We identified 122 publications across databases, and 11 additional publications through alternative routes (Fig. 1). After duplicates had been removed, 120 publications were screened based on titles and abstracts, and 33 full-text publications were reviewed. Finally, 32 publications were included. In total, 14 tools for pain in pediatric oncology were identified.

### Results scoping review

An overview of included studies and characteristics of the digital tools can be found in Table 1.

### Method of delivery

Two methods of delivery were used: apps (*n*=13, 92.9%), and a wearable wristband (*n*=1, 7.1%).

### Features

Tool features were grouped into four main themes: (1) monitoring feature for one or more pain characteristic(s) (e.g., presence, severity, perceived cause, interference with daily life, bothersomeness), (2) information about pain(management), (3) communication, and (4) game elements. In 13/14 tools, the pain characteristic “severity” was monitored (*n*=14, 92.9%), of which 12 tools used self and/or parent reports with several rating scales (details in Table 1), and one wearable tool used heart rate, skin temperature, and electrodermal activity to monitor pain [42]. One tool just assessed the pain characteristic “bothersomeness” (mouth sores, headache, hurt of pain other than headache) [43]. Information about pain(management) was provided in six tools (42.9%), and nine tools (64.3%) included a communication feature, of which *n*=3 provided real-time feedback from healthcare professionals, *n*=2 provided real-time algorithm-informed feedback, *n*=3 included a web-interface for healthcare professionals to evaluate and give feedback on data, *n*=1 included a community forum for peers, and *n*=1 provided pain reports for healthcare professionals during clinic appointments. One tool had two communication features and thus was counted twice [43]. Gamification elements were used in four tools (24.6%) and included users picking their own avatar (*n*=2), playing the role of a superhero or law-enforcer (*n*=2), having a sketch pad available (*n*=1), and reward systems for adherence to pain diary completion (*n*=2).

### End users

All tools were developed for children during cancer treatment, yet one was also available during follow-up after treatment, and one was available for children with sickle cell disease as well. Age groups of children varied, with one tool (7.1%) solely focusing on young children (ages 0–12), three tools (21.4%) on adolescents and young adults (AYAs) (ages 13–32), and ten tools (71.4%) on both young children and AYAs. With regard to setting, one tool (7.1%) could be used solely in the hospital (i.e., inpatients), five tools were meant for an outpatient setting (i.e., not during hospitalization) (35.7%), and eight tools were available for in- as well as outpatients (57.1%).

### Included studies

**Fig. 2:**
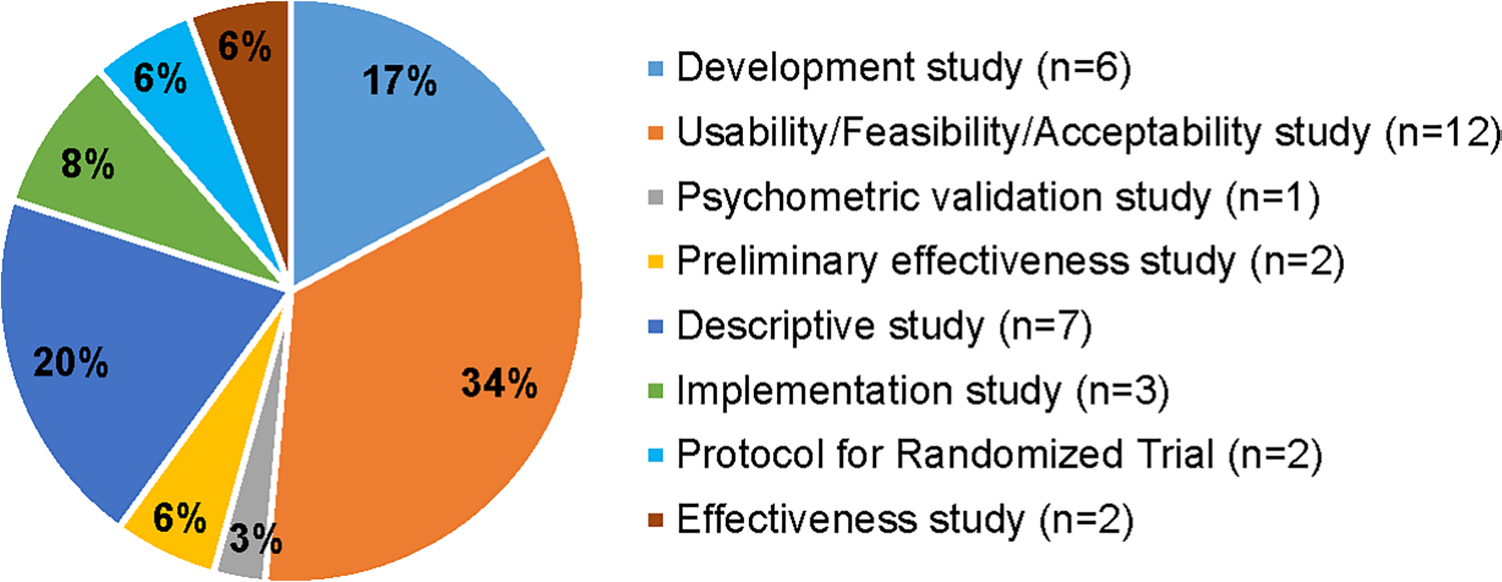
Study designs of published research (*N*=35*). *n* = number of studies per category. *33 publications were included in this review, yet two publications focused on both implementation and (preliminary) effectiveness and were counted twice in this figure

The research was published between 2012 and 2022. An overview can be found in Fig. 2. The majority of studies focused on the development and usability/feasibility/acceptability testing. For two tools (Pain Squad+ and Pain Buddy [44, 45]), preliminary data on their effectiveness in reducing pain has been published, yet no definitive results are available. For another tool (C-SCAT [46]), an effectiveness study was published. However, this study focused on the tool’s effectiveness in increasing AYA self-efficacy for symptom management, rather than pain reduction.

### Results semi-structured interviews

Thirteen project leaders were invited for a semi-structured interview on barriers and facilitators to (future) implementation of their tools (100% response rate). Table 2 describes the interviewee characteristics. For the RESPONSE app [43], no interview was conducted as this tool was added during the review process.

**Table 2:** Interviewee characteristics *(N=*13)

	***n (%)***
Gender (female)	10 (76.9)
Age (mean, range)	51.2 (39–58)
Place of residence	
2003EU	6 (46.2)
USA	5 (38.5)
Canada	2 (15.4)
Background (schooling)	
Nurse	7 (53.8)
Physician	3 (23.1)
Psychologist	2 (15.4)
Other	1 (7.7)
Years working in care (mean, range)	20.8 (min: 0, max: 37)
Years working in research (mean, range)	18.7 (min: 5, max 32)
Years working with digital health (mean, range)	12.3 (min: 2, max: 22)

*n* = number of individuals per category

A comprehensive overview of the outcomes of the semi-structured interviews (including quotes illustrating the context in which barriers and facilitators were encountered) can be found in Appendix 3.

With regard to professional input, 60.3% of the professionals were healthcare professionals (e.g., physicians,nurses,physiotherapists, psychologists, pain experts, child life specialists), 27.9% were digital technique specialists (e.g., computer scientists, engineers, software developers, applied IT specialists), and 11.8% were other professionals (e.g., lawyers, patient organization members, communication experts, measurements experts, health economists).

With regard to key stakeholders, 41.4% of all mentioned stakeholders were families (e.g., patients, parents, and extended families), 37.9% were healthcare professionals (includes the hospital as an organization), 10.3% were cancer aid organizations, 6.9% were research funders, and 3.4% were IT companies.

Five interviewees (38.5%) reported having used or using a theoretical model for implementation, namely the Consolidated Framework for Implementation Research (CFIR) (*n*=2), the Knowledge-to-Action (KTA) Framework (*n*=1), the Reach, Effectiveness, Adoption, Implementation, and Maintenance (RE-AIM) Framework (*n*=1), and the MRC (Medical Research Council) Framework for Development and Evaluation of Complex Tools (*n*=1).

**Fig. 3:**
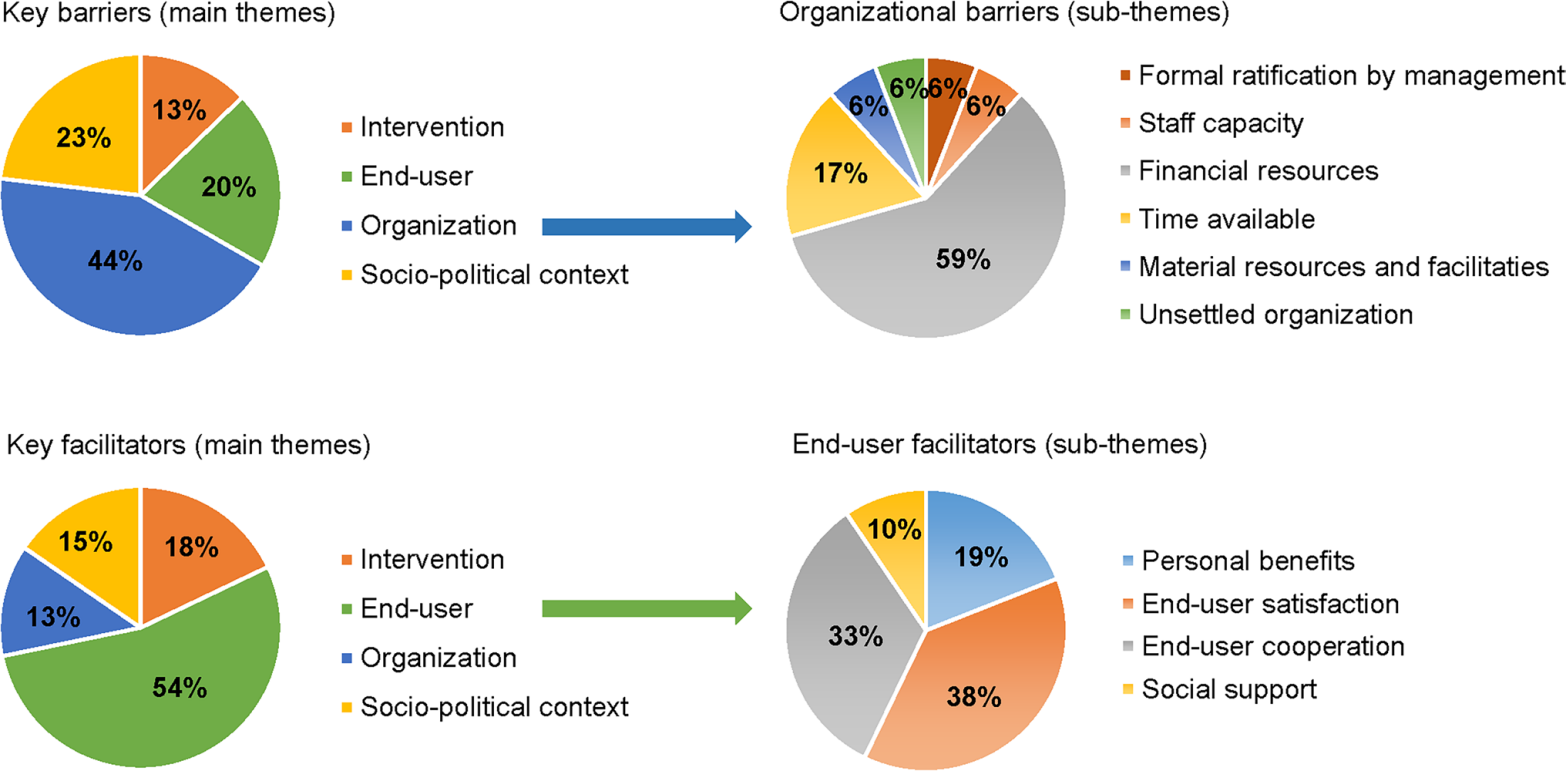
Identified key barriers and facilitators categorized in MIDI* themes and sub-themes. *MIDI: measurement instrument for determinants of innovations

The key barriers and facilitators can be found in Fig. 3. Most barriers related to the organization (i.e., financial resources: “During effectiveness testing, a nurse is paid from research funding. But how will we fund this down the road, when we want to implement/scale up?”) and the socio-political context (i.e., legislation/regulations: “When you work with different hospitals and institutions they may have different juridical regulations”). Most facilitators related to the end users (i.e., client/patient cooperation: “Children are more likely to accept new technology and to incorporate new technology into their house”) and the tool itself (i.e., complexity: “It was really easy and clear how to use the app”).

## Discussion

This review identified fourteen unique digital tools for pain monitoring, with the ultimate goal of improving pain management and reducing pain in children and/or AYAs with cancer. Identified tools in this study were mostly mobile apps that can be used in both in- and outpatient settings, by young children as well as AYAs, and were directed at pain severity monitoring using self- and/or proxy reporting rating scales. The feasibility and/or acceptability of all but one tool (RESPONSE [43]) has been established, yet very little is still known about their effectiveness in accurately monitoring and/or reducing pain [47]. Moreover, little is also known about their (future) chances of successful implementation in care. During the interviews, project leaders mostly mentioned organizational barriers and end-user facilitators in the process of implementation.

In addition to pain severity monitoring, another commonly used feature was “communication.” Seven tools included a communication feature with healthcare professionals (50% of all tools), and one tool included a community forum for communication with peers (7.1%). Communication options with healthcare professionals ranged from real-time feedback on reported pain scores, to feedback on pain scores during clinic visits (i.e., delayed feedback). A previous review on the benefits of mobile apps for cancer pain management in (mostly) adults revealed evidence for improved quality of life and decreased pain catastrophizing for digital tools with a real-time communication functionality between patients and healthcare professionals [19]. This shows promise for the future effectiveness of tools which included real-time feedback from healthcare professionals.

Most included publications focused on the development and user experiences. The biggest knowledge gap lies in these tools’ effectiveness in successfully monitoring and/or reducing pain. One effectiveness study found a significant effect on self-efficacy for symptom management in AYAs, yet no results on symptom (i.e., pain) reduction were included [46]. Two preliminary effectiveness studies on pain reduction were published and found a significant effect on pain severity (decreased) [27, 48], pain interference (decreased), and Health-Related Quality of Life (HRQOL) (increased) [27]. The tools described in these two studies (Pain Buddy and Pain Squad+) both included real-time feedback (from healthcare professionals or algorithm informed based on healthcare professionals’ input) and game elements. Game elements and in-app incentives have previously been found to increase medication adherence [49] and thus might also be useful to improve symptom reporting adherence of digital tools. However, since both studies were preliminary with small sample sizes (*N*=40/48), no definitive recommendations can be made.

A strength of this scoping review lies in the added value of the semi-structured interviews aimed at identifying key barriers and facilitators. This mixed-method design informs readers on the state of the field based on published literature but also incorporates project leaders’ experiences that may form valuable lessons for future researchers. The high response rate (100%) for interview participation in this study reflects the project leaders’ willingness to share experiences with colleagues to contribute to implementation awareness. Digital tools have the potential of being more cost-effective than regular face-to-face care [50], that is, when successfully implemented in care. A key pillar of implementation science lies in the involvement of stakeholders, and user-centered designs have previously been associated with successful implementation in care [30]. In this review, only five out of 12 interviewees reported using a theoretical model for implementation. However, all interviewees did report getting input from a diverse group of professionals (i.e., healthcare professionals, specialists in digital technique, lawyers, patient organizations) and stakeholders (i.e., families, healthcare professionals, cancer aid organizations, research funders, IT companies) throughout their projects. Healthcare professionals were by far the most involved professionals, and they were also the second most commonly mentioned key stakeholders, after families. Thus, despite the sparse use of formal theoretical models for implementation, end users’ input was highly valued as they were involved in the majority of projects. Based on the literature, this increases the chance of successful future implementation of these tools in care. The close involvement of project leaders globally in this review might lead to more international collaborations, larger sample sizes, and higher cost-effectiveness in the future. At the same time, international collaborations might cause barriers in the socio-political spectrum of the MIDI [41], such as legislation and regulations and collaborating with external stakeholders (i.e., other disciplines/hospitals/cultures).

The importance of including end users is also reflected in the results of the interviews with project leaders on barriers and facilitators. The most common facilitators were often connected to end users (56% of all mentioned facilitators), with end-user cooperation and end-user satisfaction mentioned most often. This is in line with several reviews stressing the importance of user-centered designs to accomplish successful use in routine care [25, 31–33]. In contrast to previous findings in which researchers’ intrinsic motivation (personal beliefs in the importance of making their tools available to end users) was mentioned as an important facilitator [30], this was not found in the current study. The most common barriers were identified in the organizational context (47% of all mentioned barriers), with financial resources and time available being the most common. This is in line with a previous review on digital health tools for pediatric pain (not cancer-specific) in which lack of time and infrastructure to support tool availability were identified as barriers as well [30]. The overarching aim of assessing barriers and facilitators is to identify and understand factors that influence implementation [51]. However, solely assessment of barriers and facilitators does not suffice. It is also important to act on this knowledge and focus on areas that need more attention. For this purpose, Nilsen et al. have described several models which guide the process of translating research into practice and provide more practical planning and execution of implementation endeavors [51]. Future digital health researchers should incorporate such models in their projects in order to increase implementation success.

A limitation of this study lies in the fact that the RESPONSE tool [43] did not come up in our initial literature search and was brought to our attention during the review process. As a result, we were unable to carry out the interview about barriers and facilitators. We did include this tool in Table 1 (overview tool characteristics based on published research).

This review provides an update on digital tools for acute and/or chronic pain in children with cancer that have been developed in research settings. Thirteen unique digital tools were identified, and these are mostly apps directed at pain severity monitoring. Feasibility and acceptability were established for all tools, yet definitive data on their effectiveness in accurately monitoring and/or reducing pain is lacking. Qualitative assessment of common determinants (barriers and facilitators) of successful implementation yielded valuable findings that can inform and guide future digital health researchers and implementers, not only in pediatric oncology, but also in a wide variety of both pediatric and adult healthcare populations.

## Supplementary information

APPENDIX 1. PubMed search string

APPENDIX 2. Interview guide

APPENDIX 3. Summary semi-structured interviews
