# Supplementary material for: Digital health tools for pain monitoring in pediatric oncology: a scoping review and qualitative assessment of barriers and facilitators of implementation
Source: Support Care Cancer. 2023 Feb 21;31(3):175. doi: 10.1007/s00520-023-07629-2 (PMC9944681; doi:10.1007/s00520-023-07629-2)
Supplement: Supplementary file 1 — PubMed search string [file 520_2023_7629_MOESM1_ESM.pdf]

APPENDIX 1. Included keywords and PubMed search string

The following keywords were included: mobile health, telemedicine, telehealth, mhealth, ehealth, mobile intervention, mobile application, app, smarthphone, teleconsult, pain, cancer pain, headache, migraine, neuralgic, ache, neuropathic, child, kid, youth, juvenile, pediatric, infant, schoolchild, childhood, preschooler, adolescent, teen, teenager, cancer, neoplasm, tumor, malignant, and leukemia.

("Pain"[MeSH Terms:noexp] OR "Pain"[Title/Abstract] OR "Cancer Pain"[MeSH Terms] OR "headache*"[Title/Abstract] OR "migraine*"[Title/Abstract] OR "neuralgi*"[Title/Abstract] OR "ache"[Title/Abstract] OR "aches"[Title/Abstract] OR "aching"[Title/Abstract] OR "neuropath*"[Title/Abstract])
AND
("child"[MeSH Terms] OR "child"[Title/Abstract] OR "children*"[Title/Abstract] OR "kid"[Title/Abstract] OR "kids"[Title/Abstract] OR "youth"[Title/Abstract] OR "juvenile"[Title/Abstract] OR "pediatric*"[Title/Abstract] OR "paediatric*"[Title/Abstract] OR "infant"[MeSH Terms] OR "infant*"[Title/Abstract] OR "infancy"[Title/Abstract] OR "schoolchild*"[Title/Abstract] OR "childhood"[Title/Abstract] OR "preschooler*"[Title/Abstract] OR "girl"[Title/Abstract] OR "girls"[Title/Abstract] OR "boy"[Title/Abstract] OR "boys"[Title/Abstract] OR "adolescent*"[Title/Abstract] OR "adolescent"[MeSH Terms] OR "teen"[Title/Abstract] OR "teens"[Title/Abstract] OR "teenager*"[Title/Abstract])
AND
("Neoplasms "[MeSH Terms] OR "neoplas*"[Title/Abstract] OR "tumor*"[Title/Abstract] OR "tumour*"[Title/Abstract] OR "cancer*"[Title/Abstract] OR "malignan*"[Title/Abstract] OR "leukemia*"[Title/Abstract] OR "leukaemia*"[Title/Abstract])
AND
("mobile health"[Title/Abstract] OR "Telemedicine"[MeSH Terms:noexp] OR "Telemedicine"[Title/Abstract] OR "tele medicine"[Title/Abstract] OR "telehealth"[Title/Abstract] OR "tele health"[Title/Abstract] OR "teleconsult*" OR "tele consult*"[Title/Abstract] OR "mhealth"[Title/Abstract] OR "m health"[Title/Abstract] OR "ehealth"[Title/Abstract] OR "e health"[Title/Abstract] OR "mobile intervention"[Title/Abstract] OR "mobile application"[Title/Abstract] OR "app"[Title/Abstract] OR "apps"[Title/Abstract] OR "smartphone*"[Title/Abstract])
