# Supplementary material for: Digital health tools for pain monitoring in pediatric oncology: a scoping review and qualitative assessment of barriers and facilitators of implementation
Source: Support Care Cancer. 2023 Feb 21;31(3):175. doi: 10.1007/s00520-023-07629-2 (PMC9944681; doi:10.1007/s00520-023-07629-2)
Supplement: Supplementary file 2 — Interview guide [file 520_2023_7629_MOESM2_ESM.pdf]

Interview guide

Determinants of implementation of Digital Health Interventions for Pain in Pediatric Oncology

Introduction:

“Are you ok with us recording this session as a back-up? We will not publish these recordings.
If so: could you also shortly introduce yourself?”

“We invited you to do this interview as part of a scoping review on digital health interventions for pain in pediatric oncology. We wanted to add a qualitative aspect to the review, focusing on determinants of implementation researchers encounter. Our aim with these interviews is to develop an overview with lessons for future digital health developers and researchers.”

“This interview will take up to 60 minutes, and has three sections. First, I will ask a few questions about the current status of the [intervention] project and the parties involved. Second, we will focus on which barriers and facilitators you have encountered during the [intervention] project. Third, we will talk about future plans. And finally we will ask some questions about you and your working experience.”

*Definitions of orange words can be found at the end of document.

CURRENT STATUS AND PARTIES INVOLVED

1. Please describe your role (job description) in the [intervention] project:

   Prompt: *Are you currently still working in the [intervention] project?*

2. Which phase is [intervention] currently in? For example: development, evaluation, implementation..

   Prompt: *which phase specifically: development, feasibility testing, effectiveness testing, implementation, dissemination?*

3. With regards to future implementation of [intervention], are you using OR are you planning to use an implementation theory/model/framework? And if so, which one?

   Prompt: *for example, CFIR,* PARIHS, Knowledge to Action Cycle, Theoretical Domains Framework, COM-B

4. We're wondering which professions have been involved in the project. Could you give an overview?

   Prompt: *(if not mentioned) and how about researcher(s), clinician(s), IT-specialist(s), lawyer(s), policy maker(s), economic evaluator(s), OTHER, namely:*

5. Stakeholders are people or organizations who have an interest in your research project, or affect or are affected by its outcomes. Which people or organizations are key stakeholders for your project?

   Prompt*: (if not mentioned) and how about parents, children, board of directors, healthcare professionals, researchers, OTHER, namely:*

6. Are these key stakeholders now, or have they previously been, involved in the project?

   Prompt: *please describe their involvement*

BARRIERS AND FACILITATORS

Barriers and facilitators describe factors helpful to ór hindering the development and/or implementation process of new interventions. They can play a role at different levels, namely on the intervention level, the end-user level, the organization-level or the socio-political level. To give you an idea, we have sent an overview of possible barriers and facilitators prior to the interview and asked you to look at these.

1. Which are the three major **BARRIERS** that you have encountered during the project?

- ..

  Phase: *DEVELOPMENT / FEASIBILITY TESTING / EFFECTIVENESS TESTING / IMPLEMENTATION / DISSEMINATION, OTHER:*

- ..

  Phase: *DEVELOPMENT / FEASIBILITY TESTING / EFFECTIVENESS TESTING / IMPLEMENTATION / DISSEMINATION, OTHER:*

- ..

  Phase: *DEVELOPMENT / FEASIBILITY TESTING / EFFECTIVENESS TESTING / IMPLEMENTATION / DISSEMINATION, OTHER:*

2. Which are the three major **FACILITATORS** that you have encountered during the project?

- ..

  Phase: *DEVELOPMENT / FEASIBILITY TESTING / EFFECTIVENESS TESTING / IMPLEMENTATION / DISSEMINATION, OTHER:*

- ..

  Phase: *DEVELOPMENT / FEASIBILITY TESTING / EFFECTIVENESS TESTING / IMPLEMENTATION / DISSEMINATION, OTHER:*

- ..

  Phase: *DEVELOPMENT / FEASIBILITY TESTING / EFFECTIVENESS TESTING / IMPLEMENTATION / DISSEMINATION, OTHER:*

Future plans

1. What are your future plans for the intervention?

DEMOGRAPHICS INTERVIEWEE

1. Which pronouns do you prefer?/i.e. what is your gender?
2. Age:
3. Country of residence:
4. Do you work in a hospital/university setting:
5. Clinical role: PHYSICIAN / PHARMACIST / PHYSIOTHERAPIST / NURSE / DIETITIAN / PSYCHOLOGIST / OTHER, namely:
6. Years working in clinical care:
7. Research/academic position: PROFESSOR / POST DOC / PHD STUDENT / RESEARCH ASSISTANT / OTHER, namely:
8. Years working in research:
9. Years working with digital health interventions:

Definitions

Feasibility (testing)	(Testing) the practicality of the intervention, for example in terms of learnability, usability, desirability.
Effectiveness (testing)	(Testing) the degree to which the intervention is successful in meeting the desired results.
Implementation	The process of integrating the intervention into clinical settings. / Putting interventions into use in real-world settings.
Dissemination	Effective dissemination is about actively getting the findings of your research to the people who can make use of them, to maximise the benefit of the research without delay.
Stakeholder	Stakeholders are people or organisations who have an interest in your research project, or affect or are affected by its outcomes. Stakeholders include those who are both supportive of your research, as well as those who may be less supportive or indeed critical of it (i.e. patients or healthcare professionals).
Champion	Individuals who support, market, or 'drive through' implementation in a way that helps to overcome indifference or resistance by key stakeholders (i.e. head nurse or member of patient counsil).
Implementation theory	Explains what influences implementation outcomes. Explains how or why an intervention does or does not work.
Implementation model	Describes and/or guides the process of translating research into practice. Describes the temporal sequence of implementation endeavours.
Implementation framework	A proposed model of factors/elements/determinants likely to impact implementation and sustainment of the intervention. Often includes multiple levels (system, organization, provider, patient) and phases (implementation occurs over time, often in phases).
Barrier	Prevents or hinders the intervention from further development and/or slows down the process of implementation.
Facilitator	Speeds up intervention development and/or stimulates the process of implementation.

Overview MIDI (sub)themes

Barriers/facilitators associated with the <u>innovation</u>

- Procedural clarity
- Correctness
- Completeness
- Complexity
- Compatibility
- Observability
- Relevance for client

Barriers/facilitators associated with the <u>end-user</u>

- Personal benefits/drawbacks
- Outcome expectations
- Professional obligation
- Client/patient satisfaction
- Client/patient cooperation
- Social support
- Descriptive norm
- Subjective norm
- Self-efficacy
- Knowledge
- Awareness of content of innovation

Barriers/facilitators associated with the <u>organization</u>

- Formal ratification by management
- Replacement when staff leave
- Staff capacity
- Financial resources
- Time available
- Material resources and facilities
- Coordinator
- Unsettled organisation
- Information accessible about use of the innovation

Barriers/facilitators associated with the <u>socio-political context</u>

- Legislation and regulations

Fleuren, M. A. H., Paulussen, T. G. W. M., Van Dommelen, P., & Van Buuren, S. (2014). Measurement instrument for determinants of innovations (MIDI). *Leiden: TNO*.
