# Supplementary material for: Digital health tools for pain monitoring in pediatric oncology: a scoping review and qualitative assessment of barriers and facilitators of implementation
Source: Support Care Cancer. 2023 Feb 21;31(3):175. doi: 10.1007/s00520-023-07629-2 (PMC9944681; doi:10.1007/s00520-023-07629-2)
Supplement: Supplementary file 3 — Summary semi-structured interviews [file 520_2023_7629_MOESM3_ESM.pdf]

Appendix 3. Overview outcomes semi-structured interviews

Intervention	Context research project	Stakeholder involvement	Use of implementation theory, model or framework	Identified key barriers and facilitators categorized in MIDI* main and sub-themes
**Color Me Healthy app**	• **Interviewee(s) role in project:** Principal investigator<br><br>• **Current project phase:** Second cycle of development phase<br><br>• **Professionals contributed to the project**: Nurses, nurse practitioners, physicians, specialists in videogame design, specialists in informatics	• **Key stakeholders involved:** Children, parents, nurses, nurse practitioners, physicians, child life specialists<br><br>• **During which project phase(s) were they involved?** Throughout the development of the app	No	Key barriers<br>• **Main: organization, sub: financial resources** "A really big challenge is maintaining some source of funding to keep the project going."<br>• **Main: organization, sub: time available** "Even small bugs in the code can be a setback. Having the time and dedicated team to support the work are essential."<br>• **Main: organization, sub: staff capacity** "We work with students, so their work is completed on a semester basis as opposed to having a constantly available team."<br>Key facilitators<br>• **Main: socio-political context, sub: collaborating with external stakeholders, i.e. other disciplines/hospitals/cultures** "A key facilitator is having committed interdisciplinary team-members with different perspectives."<br>• **Main: end-user, sub: cooperation** "If I don't have a parent who is supportive of working with their child in using the digital technology, then the project is less likely to be successful."<br>• **Main: end-user, sub: personal benefits** "Cloud-based data repositories can be future facilitators to access data remotely."

**C-SCAT**	• **Interviewee(s) role in project:** Principal investigator<br><br>• **Current project phase:** Seeking funding for a RCT to evaluate the efficacy of the C-SCAT to improve symptom self-management<br><br>• **Professionals contributed to the project**: Nurses, nurse scientists, computer scientists and game design experts	• **Key stakeholders involved:** AYA's<br><br>• **During which project phase(s) were they involved?** AYA's reviewed general concept for the C-SCAT while the initial grant to support the development of the C-SCAT was written	No	Key barriers<br>• **Main: socio-political context, sub: collaborating with external stakeholders, i.e. other disciplines/hospitals/cultures** "With our initial study, app development was relatively new, so we needed help identifying collaborators for programming support."<br>• **Main: end-user, sub: personal drawbacks** "Initial version proved to be overly time consuming for patients with multiple complex symptoms."<br>• **Main: end-user, sub: cooperation** "Some AYAs required more prompting than others to use their C-SCAT image in the context of a clinical visit."<br><br>Key facilitators<br>• **Main: socio-political context, sub: collaborating with external stakeholders, i.e. other disciplines/hospitals/cultures** "Access to individuals who could facilitate connections to identify collaborators for programming support."<br>• **Main: end-user, sub: satisfaction** **"**Novelty for users of interacting with a tablet computer-based app."<br>• **Main: socio-political context, sub: collaborating with external stakeholders, i.e. other disciplines/hospitals/cultures** "Multi-site collaborators to facilitate accrual of an adequate sample size."
**Empatica E4 wristband**	• **Interviewee(s) role in project:** Principal investigator<br><br>• **Current project phase:**	• **Key stakeholders involved:** Patients, parents of patients, clinicians (oncologists, hematologists), technology companies (Fitbit, Google Health)	No	Key barriers<br>• **Main: end-user, sub: compatibility** "I'm having to make the devices work for children, rather than the devices be specifically designed for children. When it comes to innovation, children aren't necessarily the clientele."

	Empatica E4 wristband has been validated in a feasibility study, patient satisfaction has been studied, next step will be effectiveness testing in a multi-site study<br><br>• **Professionals contributed to the project**:<br>Data analysts, computer scientists, physicians	• **During which project phase(s) were they involved?**<br>Patients, parents and clinicians are involved in designing the research. Technology companies provide the devices, they will be contacted while writing the grant.		• **Main: end-user, sub: satisfaction**<br>"Kids don't want to wear anything that will draw attention to them. It has got to be cool. I had to find something that records the data that I need, and fits with their personal brand."<br>• **Main: intervention, sub: correctness**<br>"The way that the medications were entered into the electrical health record were not always matched when the child received the medication."<br>Key facilitators<br>• **Main: end-user, sub: cooperation**<br>"Children are more likely to accept new technology and to incorporate new technology into their house."<br>• **Main: end-user, sub: satisfaction**<br>"The children's hospital and my colleagues were supportive of the project."<br>• **Main: intervention, sub: observability**<br>"The time stamp, and vital information was really visualized on the researcher view of the innovation"
**ePROtect**	• **Interviewee(s) role in project:**<br>Leader of the research group<br><br>• **Current project phase:**<br>Validity testing of measurement instrument used in intervention<br><br>• **Professionals contributed to the project**:	• **Key stakeholders involved:**<br>Patients, parents of patients, medical doctors and nurses, cancer aid society, the hospital<br><br>• **During which project phase(s) were they involved?**<br>Throughout the entire project	No	Key barriers<br>• **Main: organization, sub: time available**<br>"The patient needs to be educated and reminded to fill out the measurements daily. It needs to be explained why this is important."<br>• **Main: end-user, sub: cooperation**<br>"The research group works very hard on establishing new ways to measure symptoms, but the other members of the ward (e.g., medical doctors, nurses) need to be involved in order for them to accept the new ways of measurement."<br>• **Main: end-user, sub: knowledge**

	Healthcare professionals (psychologists, nurses, medical doctors), scientists, PhD students, study nurses, IT specialists			“It takes a lot of time to run the project and motivate the patients. In the phase of development and implementation it’s consuming a lot of resources.”<br>Key facilitators<br>• **Main: organization, sub: time available**<br>“The pandemic gave us more time to concentrate on this project, because we had less patients.”<br>• **Main: end-user, sub: personal benefits**<br>“The advantages that digital technique has, like the speed of time, the way of producing big data. That is a clear advantage of digital technique.”<br>• **Main: end-user, sub: personal benefits**<br>“Our web portal was not just an instrument to ask something from the patient, but it was also an instrument to give the patient a lot of information about his treatment.”
**KLIK Pain Monitor app**	• **Interviewee(s) role in project:** Project leader<br><br>• **Current project phase:** Effectiveness testing using a RCT. Depending on the outcomes, the next phase will be implementation in daily care.<br><br>• **Professionals contributed to the project**:<br>Researchers, clinicians (pediatric oncologists, psychologists, nurses	• **Key stakeholders involved:** The pain team, pediatric oncologists, nurse specialists, children and their parents<br><br>• **During which project phase(s) were they involved?**<br>Children and their parents were involved throughout the entire project. The pain team was not involved in writing the grant, because there was no pain team yet. Pediatric oncologists and	Yes: Knowledge-to-Action (KTA) Framework	Key barriers<br>• **Main: organization, sub: unsettled organization**<br>“Our organization was new when we started the project. All procedures and pathways were not clear. This includes finding a way to collaborate with the new pain team.”<br>• **Main: intervention, sub: correctness**<br>“A participant mentioned ‘I didn’t always receive the daily reminders. At one point, I didn’t receive them for two days’.”<br>• **Main: organization, sub: time available**<br>“HCP’s were worried about the extra workload.”<br>Key facilitators<br>• **Main: end-user, sub: satisfaction**

	and pain team of doctors and nurses), PhD student, IT specialists, lawyers. Educational information in the app was screened by the patient organization.	nurse specialists were involved in the feasibility study and will be involved in the implementation phase.		"A HCP mentioned 'I think that the app will increase our knowledge on how often kids are in pain at home. And it enables us to provide them with care much quicker'."<br>• **Main: end-user, sub: satisfaction**<br>"A patient mentioned 'It worked perfectly: customer friendly, intuitively, simple. I didn't experience any problems'."<br>• **Main: intervention, sub: complexity**<br>"It was really easy and clear how to use the app."
**Kræftværket app**	• **Interviewee(s) role in project:**<br>Project owner, project planner and head of research group<br>• **Current project phase:**<br>The app is implemented and disseminated throughout Denmark as a research project. Currently working on implementation in daily clinical care.<br>• **Professionals contributed to the project**:<br>Researchers, clinicians, software developers, specialists in interface development, doctors (oncologists, pediatricians, hematologists), nurses, medical students, experts in communication, social workers, psychologists	• **Key stakeholders involved:**<br>Patients, national network for AYA's with cancer (Danish Cancer Society)<br>• **During which project phase(s) were they involved?**<br>They are in the steering committee of the project (involved throughout project)	No	Key barriers<br>• **Main: socio-political context, sub: legislation and regulations**<br>"When you work with different hospitals and institutions they may have different juridical regulations."<br>• **Main: organization, sub: financial resources**<br>"It is difficult to get funding."<br>• **Main: end-user, sub: social support**<br>"When you developed an intervention in one institution than you might face the challenge that others are not willing to take ownership for that e-Health tool. 'How do you know that it works at our institution?'."<br>Key facilitators<br>• **Main: end-user, sub: cooperation**<br>"This patient population feels the need to do something for others."<br>• **Main: organization, sub: coordinator**<br>"Someone who is willing to move around, who is not only in the office phoning and writing to everyone, but also goes out to other hospitals, listen to how they work there, try to work with them and help them with implementation."<br>• **Main: organization, sub: financial resources**

				"When we had good financing, the project went well. At times when we had less funding, it was difficult."
**mOST** (Mobile Oncology Symptom Tracker)	• **Interviewee(s) role in project:** Co-project leader<br><br>• **Current project phase:** The project has been terminated after the usability/acceptability study, because the project leader had to decide which intervention would be developed further and decided to focus on another intervention.<br><br>• **Professionals contributed to the project**: Research nurses, physicians and IT specialists	• **Key stakeholders involved:** Clinicians (physicians, nurses and pharmacists) involved in care<br><br>• **During which project phase(s) were they involved?** After usability/acceptability study	No	Key barriers<br>• **Main: organization, sub: formal ratification by management** "Getting approval from our contract office, because we worked with an outside group which the university never worked with before. This took months and months and months."<br>• **Main: intervention, sub: procedural clarity** "I struggled to make the intervention visually appealing and uniform looking when we were using different measurement tools."<br>• **Main: organization, sub: replacement when staff leave** "The contract had been signed with the university where I was and I was leaving to another university. I could not take the project with me."<br>Key facilitators<br>• **Main: end-user, sub: cooperation** "Kids wanted to have an iPhone so at the start of the project this was a facilitator."<br>• **Main: organization, sub: financial resources** "We had the financial resources to use money as incentives for the participants."<br>• **Main: intervention, sub: complexity** "The simplicity of the intervention was a facilitator, it was very quick to answer the questions."
**OPBG** (Ospedale Pediatrico	• **Interviewee(s) role in project:** Project leader	• **Key stakeholders involved:** The hospital, patients, committee for pain	No	Key barriers<br>• **Main: intervention, sub: compatibility** "Due to it being 2014, not everyone had a smartphone."

Bambino Gesu Tool)	• **Current project phase:** Development phase is completed. App was used only in the research project, might be used in the future again. Open to continuing its' development.<br><br>• **Professionals contributed to the project**: App developer ('IT nurse'), researchers, research nurses, pediatric oncologist, medical lawyers	• **During which project phase(s) were they involved?** During first study		• **Main: socio-political context, sub: legislation and regulations** "We developed an app, but now another department within the hospital deals with applications. In order to use it now, it has to go through them."<br>• **Main: organization, sub: material resources and facilities** "Even the platforms to develop the app weren't as easy accessible as they are now. You needed more knowledge to develop an app than you need now."<br>Key facilitators<br>• **Main: intervention, sub: procedural clarity** "We already had certain standards for pain evaluation, we had a clear structure to base our work on."<br>• **Main: end-user, sub: satisfaction** "We perceived a 100% satisfaction."<br>• **Main: end-user, sub: social support** "The other nurses helped us find patients, they knew when patients would be discharged so we could contact them about participation in the study."
**Pain Buddy**	• **Interviewee(s) role in project:** Principal Investigator<br><br>• **Current project phase:** Ongoing effectiveness study (RCT). Meanwhile conducting implementation interviews with children, families and HCP's to increase implementation effectiveness. Working on cultural	• **Key stakeholders involved:** Clinicians, children and their parents, the hospital<br><br>• **During which project phase(s) were they involved?** Clinicians were involved throughout the process and have co-designed the intervention with us. Throughout each phase	Yes: Reach, Effectiveness, Adoption, Implementation, and Maintenance (RE-AIM) Framework	Key barriers<br>• **Main: socio-political context, sub: collaborating with external stakeholders, i.e. other disciplines/hospitals/cultures** "As academic researchers we are used to the process evolving over time, or realizing 'Wait we need this feature', especially if you have stakeholder input. Whereas the developers want everything at the outset and then you are locked to that. The communication was challenging as well."<br>• **Main: socio-political context, sub: collaborating with external stakeholders, i.e. other disciplines/hospitals/cultures**

	adaptation, social support components and parent component in the app.<br><br>• **Professionals contributed to the project**:<br>Clinicians (oncology nurses, nurse practitioners, bachelor and master level nurses, oncology physicians, child life specialists, anesthesiologists and psychologists), computer scientists, engineers, people with public health background	children and their parents were involved in the design, look and function of the intervention.		"There has been a lot of hiccup's in technology that we can not fix. Relying on other teams to make those fixes has been a challenge."<br>• **Main: organization, sub: financial resources**<br>"Development of a digital intervention over years is costly, mobile health apps can be difficult to fund."<br><u>Key facilitators</u><br>• **Main: end-user, sub: cooperation**<br>"We used feedback from end-users to gamify the app, which helped a lot."<br>• **Main: end-user, sub: personal benefits**<br>"The HCP's needed to be convinced that the app would decrease their workload. It was really helpful when they saw that it actually facilitated their work."<br>• **Main: end-user, sub: satisfaction**<br>"Parents tell us that they really like it and that they want to be more integrated into it."
**Pain Squad app**	• **Interviewee(s) role in project:**<br>Project leader<br><br>• **Current phase of intervention:**<br>Dissemination/scaling up<br><br>• **Professionals contributed to the project**:<br>Healthcare professionals (pediatric oncologists, nurses, physiotherapists, psychologists,	• **Key stakeholders involved:**<br>Patients, families of patients, healthcare professionals, research funders<br><br>• **During which project phase(s) were they involved?**<br>Development, testing, and implementation	Yes: Consolidated Framework for Implementation Research (CFIR)	<u>Key barriers</u><br>• **Main: organization, sub: financial resources**<br>"During effectiveness testing, a nurse is paid from research funding. But how will we fund this down the road, when we want to implement/scale up?"<br>• **Main: socio-political context, sub: legislation and regulations**<br>"Interventions like these need to tick a lot of boxes to ensure that it complies with a certain quality and (data) safety. How will we get the app certified for use in clinical care?"<br>• **Main: organization, sub: financial resources**<br>"Apps need constant software updates, and the look and feel of app

	pain experts, child life specialists), software developers, measurement experts			need to be updated regularly as well. How will we be able to pay for this beyond research funding?"<br>Key facilitators<br>• **Main: intervention, sub: compatibility**<br>"By repeated testing, we were able to tick all boxes and remove barriers of use of the app."<br>• **Main: end-user, sub: cooperation**<br>"The ultimate goal is to have staff prescribe the app to patients, and have patients use it. Therefore, their input during development and testing phases is essential to develop a compatible intervention they support."<br>• **Main: socio-political context, sub: collaborating with external stakeholders, i.e. other disciplines/hospitals/cultures**<br>"Is it essential to work with software companies who speak the same language. Companies with strictly commercial interests often do not."
**Pain Squad + app**	• **Interviewee(s) role in project:** Project leader<br>• **Current phase of intervention:** Effectiveness testing<br>• **Professionals contributed to the project**: Healthcare professionals (pediatric oncologists, nurses, physiotherapists, psychologists, pain experts, child life specialists),	• **Key stakeholders involved:** Patients, families of patients, healthcare professionals, research funders<br>• **During which project phase(s) were they involved?** Development, testing, and implementation	Yes: Consolidated Framework for Implementation Research (CFIR)	Key barriers<br>• **Main: organization, sub: financial resources**<br>"During effectiveness testing, a nurse is paid from research funding. But how will we fund this down the road, when we want to implement/scale up?"<br>• **Main: socio-political context, sub: legislation and regulations**<br>"Interventions like these need to tick a lot of boxes to ensure that it complies with a certain quality and (data) safety. How will we get the app certified for use in clinical care?"<br>• **Main: organization, sub: financial resources**<br>"Apps need constant software updates, and the look and feel of app need to be updated regularly as well. How will we be able to pay for this beyond research funding?"

	software developers, measurement experts			Key facilitators<br>• **Main: intervention, sub: compatibility**<br>"By repeated testing, we were able to tick all boxes and remove barriers of use of the app."<br>• **Main: end-user, sub: cooperation**<br>"The ultimate goal is to have staff prescribe the app to patients, and have patients use it. Therefore, their input during development and testing phases is essential to develop a compatible intervention they support."<br>• **Main: socio-political context, sub: collaborating with external stakeholders, i.e. other disciplines/hospitals/cultures**<br>"Is it essential to work with software companies who speak the same language. Companies with strictly commercial interests often do not."
**PicPecc** (Pictorial support in person-centred care for children)	• **Interviewee(s) role in project:** Principal Investigator<br><br>• **Current project phase:** Previous phase: assessing needs of children, their parents and HCP's Validity testing of measurement tool (Faces Thermometer Scale), effectiveness study of tool usage during chemotherapy, study which assesses the needs of children which could be implemented into the app. Our main goal is international collaboration, but we	• **Key stakeholders involved:** Children, their parents, HCP's, childhood cancer organization<br><br>• **During which project phase(s) were they involved?** Development phase	Yes: MRC (Medical Research Council) Framework for Development and Evaluation of Complex Interventions	Key barriers<br>• **Main: organization, sub: financial resources**<br>"We can find finding to create interventions, but it's harder to convince the clinic / funders to pay for the support and further development of an already created app. This is a management problem, because the nurses and physicians are cooperating but the funding is decided on higher levels."<br>• **Main: end-user, sub: social support**<br>"There is a gap between the academic and the clinic. So once you have developed an intervention in the university setting, you don't necessarily have buy-in or acceptance of the intervention at the hospital."<br>• **Main: socio-political context, sub: collaborating with external stakeholders, i.e. other disciplines/hospitals/cultures**

	are still applying for money to make that happen.<br><br>• **Professionals contributed to the project**:<br>Physicians (pediatric oncologists), speech/language therapists, pedagogical researchers (educational sciences), applied IT specialists, nurses, physiotherapists, dentists, health economists			"A lot of similar interventions are developed in different countries. An international network is lacking."<br>Key facilitators<br>• **Main: end-user, sub: satisfaction**<br>"We have a user-centered design, so a facilitator is patient satisfaction."<br>• **Main: socio-political context, sub: collaborating with external stakeholders, i.e. other disciplines/hospitals/cultures**<br>"Because HCP's from different disciplines were involved, we were able to create an app which is usable in different fields."<br>• **Main: socio-political context, sub: collaborating with external stakeholders, i.e. other disciplines/hospitals/cultures**<br>"We collaborated with South Africa from the beginning. We have the international perspective as well, because we started building in eleven languages in the app from the beginning."
**Telemonitoring System for Paediatric Oncology**	• **Interviewee(s) role in project:**<br>Supervisor of master student who developed the app and project manager<br><br>• **Current project phase:**<br>Previous phases: development phase and feasibility phase<br>Current phase: trying to get funding in order to further develop based on feasibility study	• **Key stakeholders involved:**<br>Patients, parents, nurses, mobile team (HCP's, such as pediatric oncologists, who visit patients)<br><br>• **During which project phase(s) were they involved?**<br>Feasibility testing	No	Key barriers<br>• **Main: socio-political context, sub: legislation and regulations**<br>"You need a medical device certification, which is doable, but it takes a lot of time and money."<br>• **Main: end-user, sub: social support**<br>"You need to involve everybody who's workflows will change because otherwise the project won't work."<br>• **Main: organization, sub: financial resources**<br>"So far, all we did was with our own money, we have some money we can use, but at some point we have to get some funding."<br>Key facilitators<br>• **Main: end-user, sub: satisfaction**

	• **Professionals contributed to the project**:<br>Researchers, IT specialists, master student, physicians, specialists in pediatric oncology, data managers, nurses			"Usability is extremely important. The usability was quite well and well accepted, people liked it quite lot."<br>• **Main: organization, sub: staff capacity**<br>"It is extremely important to have somebody who is open and able to talk to the patients, parents and physicians in the right way."<br>• **Main: end-user, sub: social support**<br>"You need to involve everybody who's workflows will change and get everybody involved because otherwise the project won't work."

Note. MIDI: Measurement Instrument for Determinants of Innovations
